# Delayed circadian rhythms in older Africans living with human immunodeficiency virus (HIV)

**DOI:** 10.1111/jpi.12838

**Published:** 2022-11-06

**Authors:** Kirsten N. Redman, Katie E. O'Brien, Francieli S. Ruiz, Dale E. Rae, F. Xavier Gómez‐Olivé, Malcolm von Schantz, Karine Scheuermaier

**Affiliations:** ^1^ Wits Sleep Laboratory, Brain Function Research Group, School of Physiology, Faculty of Health Sciences University of the Witwatersrand Johannesburg South Africa; ^2^ Faculty of Health and Medical Sciences University of Surrey Guildford Surrey UK; ^3^ Department of Human Biology, Health through Physical Activity, Lifestyle and Sport Research Centre & Division of Physiological Science, Faculty of Health Sciences University of Cape Town Cape Town South Africa; ^4^ Medical Research Council/Wits Rural Public Health and Health Transitions Research Unit (Agincourt), School of Public Health, Faculty of Health Sciences University of the Witwatersrand Johannesburg South Africa; ^5^ Faculty of Health and Life Sciences Northumbria University Newcastle‐upon‐Tyne UK

**Keywords:** circadian rhythm disorders, human immunodeficiency virus, neuroendocrinology

## Abstract

The increasing number of people living with human immunodeficiency virus, HIV, (PLWH) have an elevated incidence of risk for noncommunicable comorbidities, the aetiology of which remains incompletely understood. While sleep disturbances are often reported in PLWH, it is unknown to what extent they relate to changes in the circadian and/or sleep homeostatic processes. We studied the relationship between sleep characteristics, circadian phase, and HIV status in older adults from the HAALSI (Health and Ageing in Africa: a Longitudinal Study of an INDEPTH Community in South Africa) subsample of the Agincourt Health and Demographic Surveillance System in South Africa (*n* = 187, 36 human immunodeficiency virus positive [HIV+], age: 66.7 ± 11.5 years, range 45—93 years), where HIV prevalence is high and (in contrast to the global north) does not associate significantly with potentially confounding behavioural differences. In participants with valid actigraphy data (*n* = 172), regression analyses adjusted for age and sex indicated that HIV+ participants had slightly later sleep onset (*β* = .16, *p* = .039), earlier sleep offset times (*β* = −.16, *p* = .049) and shorter total sleep times (*β* = −.20, *p* = .009) compared to the HIV negative (HIV−) participants. In a subset of participants (*n* = 51, 11 HIV+), we observed a later dim light melatonin onset (DLMO) in HIV+ (21:16 ± 01:47) than in HIV− (20:06 ± 00:58) participants (*p* = .006). This substantial difference remained when adjusted for age and sex (*β* = 1.21; *p* = .006). In 36 participants (6 HIV+) with DLMO and actigraphy data, median phase angle of entrainment was −6 min in the HIV+ group and +1 h 25 min in the HIV− group. DLMO time correlated with sleep offset (*ρ* = 0.47, *p* = .005) but not sleep onset (*ρ* = −0.086, *p* = .623). Collectively, our data suggest that the sleep phase occurred earlier than what would be biologically optimal among the HIV+ participants. This is the first report of a mistimed circadian phase in PLWH, which has important potential implications for their health and well‐being, especially given the well‐established relationships between circadian asynchrony and sleep deprivation with poorer health outcomes.

## 1 INTRODUCTION

The development and global roll‐out of antiretroviral therapy (ART) has dramatically increased the number of people living with human immunodeficiency virus (HIV) (PLWH). Calculated life expectancy at 20 years of age for people receiving ART ranges from 60% of normal (in Rwanda) to 89% (in Canada).^1^ Improved odds for long‐term survival result in a growing ageing population of PLWH. In the United States, nearly half of PLWH are now 50 years or older.^2^ By necessity, the systems for caring for PLWH were initially designed to manage and treat primary and secondary infections. Consequently, these systems are not necessarily optimised and integrated for managing prevention and treatment of noncommunicable comorbidities in people who are living with well‐controlled HIV infection for years or decades.^3^ Prominent among noninfectious comorbidities in PLWH are cardiometabolic disorders.^4^, ^5^ PLWH have a twofold higher risk of developing cardiovascular disease (CVD) than uninfected individuals.^6^ While conditions such as hypertension, hypercholesterolaemia, and diabetes can be treated pharmacologically, modification of behaviours such as diet, physical activity and sleep are also important strategies to prevent and manage these disorders. Poor sleep quality is a well‐established consequence of living with HIV, with an estimated prevalence of around 58%.^7^ While generalised sleep disturbances are widely reported in PLWH, the exact nature of these disturbances is scarcely discussed in the existing literature and generally lacks biological precision.^8^ Reports describe cognitive performance decrements associated with higher daytime sleepiness,^9^ symptoms associated with insomnia^10^ and atypical, apparently weight‐independent presentations of obstructive sleep apnoea^11^ in PLWH. Additionally, in a cohort of veterans, it was observed that PLWH with ‘highly bothersome symptoms of insomnia’ had a higher risk of incident CVD than PLWH without insomnia, suggesting an independent role of sleep in enhancing CVD risk in PLWH.^12^

Thus far, research on sleep in HIV has primarily focused on its homeostatic components (as measured through sleep duration and staging) rather than testing the hypothesis that HIV may also impact circadian‐related aspects of sleep (e.g., sleep–wake timing) or core measures of circadian regulation. There are some suggestions in the literature that HIV may indeed be associated with changes in circadian regulation. In a mouse model, intraparenchymal injection of HIV Tat protein into the suprachiasmatic nucleus (SCN, the master circadian pacemaker) was associated with phase delay shifts in the rest–activity rhythms.^13^ In one human study, PLWH had higher plasma melatonin levels in the morning (09:00) than HIV‐negative (HIV−) individuals, suggesting a possible delay in the circadian phase, and higher morning melatonin concentration was associated with higher concomitant levels of Tat protein.^14^ Disrupted circadian rhythms in PLWH could at least partially explain the poor sleep quality and associated daytime dysfunction noted in this population, a likely risk factor for the increased prevalence of cardiometabolic comorbidities.

South Africa has the fourth highest prevalence of HIV globally (14%).^15^ It also has one of the largest ART programmes,^16^ which has enabled a decreased mortality and increased life expectancy. This high HIV prevalence in the community provides a unique opportunity (especially in rural areas) to investigate associated research questions within the traditional demographic composition and behavioural risk factors associated with HIV infection in the global north (e.g., gay men, other men who have sex with men and people who inject drugs, which is a well‐documented disruptor of circadian rhythms^17^) being much less apparent. These unique circumstances enable the investigation of the biological associations between HIV infection, sleep, and circadian rhythms in a way that is not confounded by behavioural risk factors. Second, the rural setting of the study conveys the benefit of a stronger relationship with natural Zeitgebers (with lower availability of artificial lighting) and potentially reduces confounding factors associated with urban lifestyle.^18^, ^19^

Thus, the aim of this study was to compare objectively measured aspects of the circadian phase and actigraphy‐derived sleep characteristics between older, rural individuals living with HIV to those who are HIV−.

## 2 MATERIALS AND METHODS

### 2.1 Participants and study design

**Figure 1:**
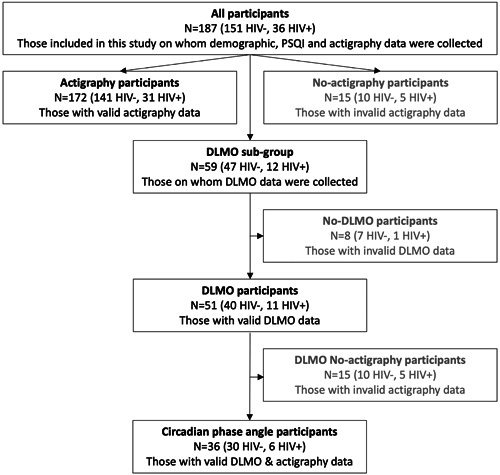
Diagram showing sample sizes and HIV status composition of all participants in this study as well as subgroups for actigraphy, DLMO and circadian phase angle analyses. DLMO, dim light melatonin onset; HIV, human immunodeficiency virus; PSQI, Pittsburgh sleep quality index.

Participants in this study were a random sample representative of the HAALSI (Health and Ageing in Africa: a Longitudinal Study of an INDEPTH Community in South Africa) study, which in turn is a representative sample of participants 40 years and above within the Agincourt Health and Socio‐demographic Surveillance System.^20^ This investigation conformed with the tenets of the Declaration of Helsinki and was approved by the University of the Witwatersrand Human Research Ethics Committee (#M180667) and the Mpumalanga Provincial Research and Ethics Committee. Written informed consent was obtained from all participants. For illiterate participants, a witness not linked to the study was present during the consenting process and signed and dated the form on behalf of themselves and the participant. This rural cohort is based in the Bushbuckridge subdistrict, Mpumalanga Province, South Africa (24.4°S 31.5°E). We present preliminary data for a subgroup of participants from a larger study.^21^ Figure 1 displays the sample sizes for all participants along with groups that explored actigraphy, dim light melatonin onset (DLMO), and circadian phase angle data in the present study. Demographic (age, sex, employment status, wealth index), Pittsburgh Sleep Quality Index (PSQI)^22^ and actigraphy data were collected for 187 participants (HIV−: *n* = 151, 81%; HIV+: *n* = 36, 19%). Of these, 172 participants (age range: 45–93 years) had valid actigraphy data (HIV−: *n* = 141, 82%; HIV+: *n* = 31, 18%). A subgroup of 59 individuals participated in a protocol to determine DLMO, HIV−: *n* = 47, 80%; HIV+: *n* = 12, 20%). Valid DLMO data were available for 51 of these participants (HIV−: *n* = 40, 78%; HIV+: *n* = 11, 22%). Finally, the circadian phase angle of entrainment was calculated from a subgroup of 36 participants with both valid actigraphy and DLMO data (HIV−: *n* = 30, 84%; HIV+: *n* = 6, 16%).

### 2.2 HIV status

HIV status was determined using the dried blood spot (DBS) assay. Five blood drops were collected from each participant using the finger prick technique on Whatman 903 TM filter paper and kept at room temperature for 1–3 weeks. The DBS was sent to Global Labs and stored at −20°C until the subsequent Vironostika Uniform 11 (Biomerieux) immunoassay. If positive, confirmation was performed using Elecsys (Roche). HIV+ status based on immunoreaction was then confirmed by reverse transcription‐polymerase chain reaction‐based viral load analysis.

### 2.3 Actigraphy

Participants wore an accelerometer (ActTrust; Condor Instruments) on their nondominant wrist for 14 consecutive days. Files were processed in ActStudio (Condor Instruments) and visually inspected to ascertain data quality. Sleep onset (time at which sleep started) and offset (time at which sleep ended) were manually marked according to published actigraphy protocols.^23^ Subsequently, application of the Cole‐Kripke algorithm in ActStudio generated estimates of sleep‐based variables, including sleep onset and offset, wake after sleep onset (WASO), total sleep time (time elapsed between sleep onset and sleep offset, less WASO) and sleep efficiency (percent time spent asleep between sleep onset and offset). Each of these variables was averaged across all valid days (minimum of 5 consecutive nights) of actigraphy data for each participant. Because actigraphy data were invalid for 15 participants (including 5 HIV+), we only present actigraphy data for 172 participants (HIV+: *n* = 31, 18%; Figure 1).

### 2.4 DLMO

Saliva samples were collected hourly between 17:00 and 23:00 while participants sat in a dimly lit (<3 lux) room at the laboratory at Agincourt. Participants were asked to accumulate saliva in their mouths and spit directly into 15‐ml collecting tubes, as described previously.^18^ Saliva was stored at −20°C until subsequent analysis at the University of the Witwatersrand. We were able to limit the melatonin assay to six samples per person by excluding one collected sample from the radioimmunoassay. Using the usual relationship between DLMO and bedtime (DLMO occurring ~2 h before bedtime), we used the participant's known bedtime as an approximation to decide whether we should rather assay the earlier sample (17:00) or the later sample (23:00). Saliva was stored at −20°C until subsequent analysis at the University of the Witwatersrand. Samples were thawed and centrifuged to remove any debris. The supernatant was processed to determine melatonin saliva concentration using the direct saliva melatonin radioimmunoassay kit (Bühlmann Laboratories) (intraassay precision: 2.6%–20.1%; interassay precision: 6.6%–16.7%). Each sample was assayed in duplicate. The standard curve for each melatonin kit was determined using Prism 9 Software (GraphPad) and melatonin concentration for each participant was computed based on the standard curve for each kit. For participants whose measurements did not reach the 3 pg/ml cutoff for DLMO but had a clear rising slope of melatonin secretion, we extrapolated up to the 3 pg/ml limit to determine DLMO time. We only did this when the 3 pg/ml threshold was estimated to happen in the hour following the last sampled melatonin, assuming the slope remained constant between the last hour of sampling and this unsampled hour. Thus, we used the slope (change in melatonin concentration over 1 h) of the previous hour and calculated the time required to get from the last sample to reach 3 pg/ml. Of the 59 participants with melatonin data, eight had no detectable levels of melatonin during the sampling period and were labelled nonsecretors. We therefore present DLMO analysis data for 51 participants (HIV+: *n* = 11, 21.6%; Figure 1). Finally, we determined the circadian phase angle of entrainment for a subset of participants who had both valid actigraphy and DLMO data (*n* = 36, HIV+: *n* = 6; Figure 1). To do this, we subtracted DLMO time from habitual sleep onset time obtained from actigraphy. For the majority of participants, wherever possible, actigraphy immediately followed the DLMO collection.

### 2.5 Data and statistical analyses

Data are presented as mean ± SD, count (percentage) and *β*‐coefficient (95% confidence intervals). Between‐group (HIV− vs. HIV+) comparisons of our demographic, actigraphy and DLMO variables of interest were made using analysis of variance (ANOVA) or chi‐squared tests as appropriate. Spearman's correlation was used for bivariate associations. General linear models (GLMs) were constructed to assess the relationship between HIV status and DLMO/collected actigraphy parameters, controlling for age and sex. For statistically significant models, the effect size was established through the calculation of partial eta‐squared (denoted by *η* ^2^_p_). Data were analysed using RStudio.

## 3 RESULTS

Descriptive demographic, health and sleep characteristics and unadjusted univariate analyses of the HIV− and HIV+ groups are presented in Table 1. HIV+ participants were younger (*p* < .001), had lower body mass index (*p* = .049), were less likely to be hypertensive (*p* = .011) and had lower total sleep time (*p* = .035) than the HIV− participants.

**Table 1:** Demographic, health and sleep characteristics of all participants

	HIV− (*N* = 151)	HIV+ (*N* = 36)	Total (*N* = 187)	*p* Value
Age (years)	68.2 ± 11.2	60.6 ± 10.6	66.7 ± 11.5	**<.001**
Sex				.693
Female	87 (59.2%)	20 (55.6%)	107 (58.5%)	
Male	60 (40.8%)	16 (44.4%)	76 (41.5%)	
BMI (kg/m^2^)	26.9 ± 9.0	24.7 ± 9.8	26.5 ± 9.2	**.049**
Hypertension	89 (59.3%)	11 (34.4%)	100 (54.9%)	**.011**
Diabetes	9 (6.0%)	0 (0.0%)	9 (4.9%)	.366
Employment status				.158
Employed	16 (10.7%)	8 (22.2%)	24 (13.0%)	
Homemaker	15 (10.1%)	3 (8.3%)	18 (9.7%)	
Unemployed	118 (79.2%)	25 (69.4%)	143 (77.3%)	
Wealth quintile	3.2 ± 1.5	2.9 ± 1.4	3.3 ± 1.5	.246
PSQI global score	4.0 ± 3.2	4.7 ± 3.5	4.1 ± 3.3	.234

Actigraphy data	HIV− (*N* = 141)	HIV+ (*N* = 31)	Total (*N* = 172)	*p* Value
Sleep onset (hh:mm)	21:00 ± 01:01	21:27 ± 01:05	21:04 ± 01:03	.109
Sleep offset (hh:mm)	06:02 ± 00:56	05:40 ± 01:28	05:58 ± 01:03	.337
Total sleep time (hh:mm)	07:10 ± 01:31	06:30 ± 01:15	07:03 ± 01:29	**.035**
WASO (hh:mm)	01:45 ± 00:42	01:41 ± 00:36	01:45 ± 00:41	.723
Sleep efficiency (%)	78.9 ± 8.1	77.9 ± 5.9	78.7 ± 7.8	.385

*Note*: Data are presented as unadjusted mean ± SD or count (percentage). Between‐group comparisons were performed using ANOVA or chi‐squared tests. Bold values Indicate statistical significance.

Abbreviations: ANOVA, analysis of variance; BMI, body mass index; HIV, human immunodeficiency virus; PSQI, Pittsburgh sleep quality index; WASO, wake after sleep onset.

### 3.1 Actigraphy‐derived sleep characteristics

Regression models, adjusted for age and sex, assessing associations between HIV status and the sleep variables obtained from actigraphy are summarised in Table 2. We found suggestive evidence for a slightly later sleep onset (adjusted average delay of 0.16 h, i.e., 10 min; *F*(3,168) = 1.63; *p* = .039) and earlier sleep offset (adjusted average advance of 0.16 h, i.e., 10 min; *F*(3,168) = 2.65; *p* = .049) in HIV+ participants compared to HIV− participants when adjusting for age and sex. Additionally, total sleep time in HIV+ participants was shorter compared to HIV− participants when adjusting for age and sex (adjusted average total sleep time of 0.20 h, i.e., 12 min; *F*(3,168) = 2.69; *p* = .009; observed power = 0.625, *η* ^2^_p_ = 0.03). No significant relationships were observed between HIV status and WASO or sleep efficiency.

**Table 2:** Standardised *β*‐coefficients extracted from linear regression models to investigate the relationships between actigraphy‐derived sleep characteristics and HIV status (*n* = 172)

Variable	Sleep onset	Sleep offset	Total sleep time	WASO	Sleep efficiency
Model number	(1)	(2)	(3)	(4)	(5)
HIV+	0.16* (0.22)	−0.16* (0.22)	−0.20 (0.31)**	−0.01 (8.44)	−0.08 (1.59)
Age (years)	−0.01 (0.01)	−0.11 (0.01)	−0.13 (0.01)	0.11 (0.28)	−0.14 (0.05)
Sex—male	0.02 (0.16)	−0.02 (0.16)	0.00 (0.23)	−0.03 (6.37)	−0.00 (1.21)

*Note*: Data are presented as *β*‐coefficients (95% confidence interval).

Abbreviations: HIV, human immunodeficiency virus; WASO, wake after sleep onset.

* *p* < .05

** *p* < .01.

### 3.2 DLMO

**Figure 2:**
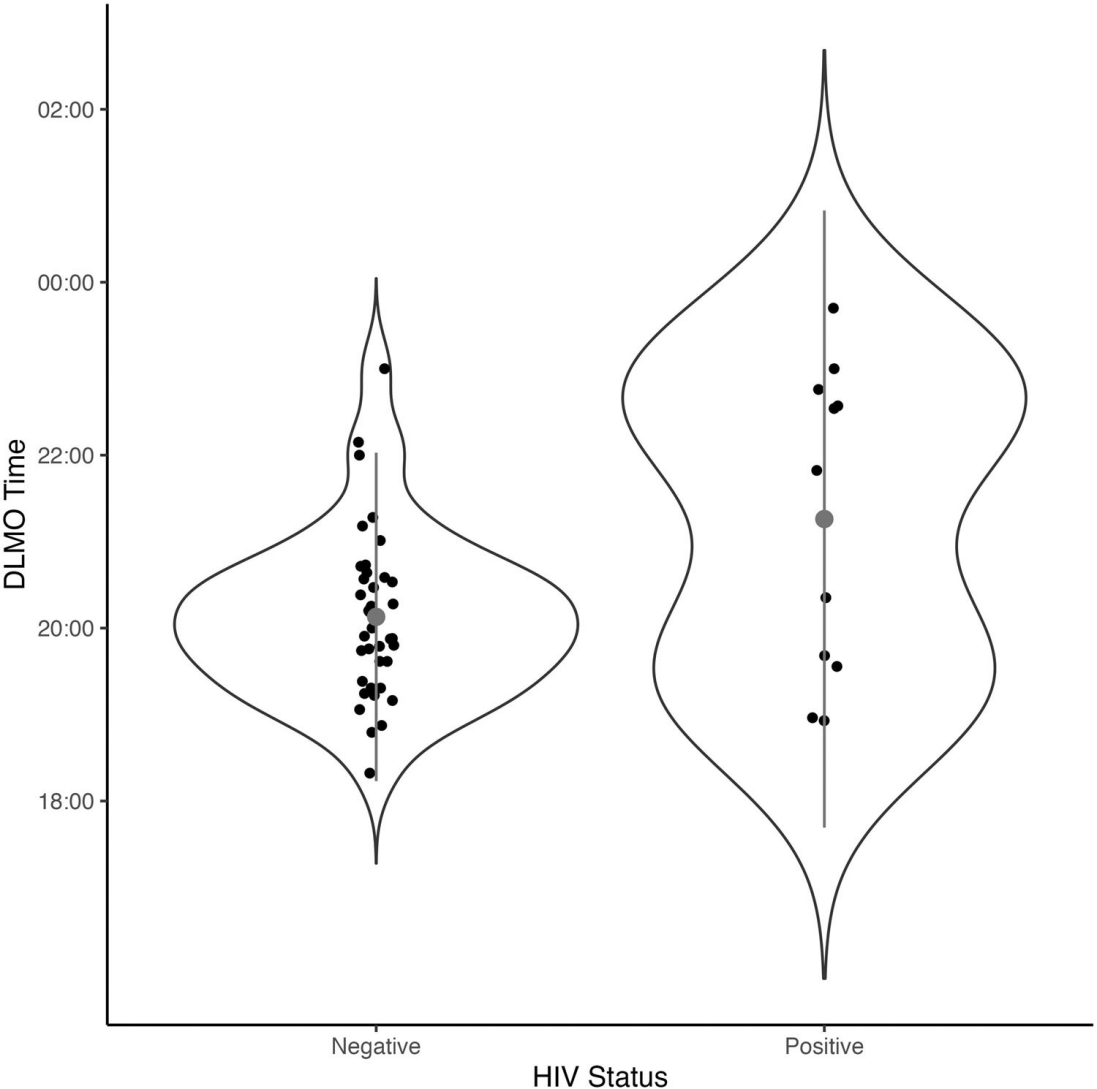
Timing of DLMO in HIV− (*n* = 40) and HIV+ (*n* = 11) participants. Individual data points are plotted in addition to the median represented by the larger grey dot, with the dispersion displayed as a violin plot. **A statistical between‐group difference (*p* = .006) determined using one‐way ANOVA. ANOVA, analysis of variance; DLMO, dim light melatonin onset; HIV, human immunodeficiency virus.

Age (64.2 ± 7.6 years) and gender distribution (32 females, 63%) of the subset of participants for whom DLMO data were available (*n* = 51, HIV+: *n* = 11) were not different to the larger sample. There were no significant relationships between DLMO times and either age, sex, employment status, PSQI score, or wealth quintile (data not shown). HIV+ participants had significantly later DLMO times compared to HIV− participants (HIV−: 20:06 ± 00:58, HIV+: 21:16 ± 01:47; one‐way ANOVA *F*(1, 49) = 8.46, *p* = .006) (Figure 2). As the average age of the HIV+ participants was lower than that of the HIV− participants, we also ran a GLM on the effect of HIV status on DLMO time when adjusting for age and sex. This confirmed a large significant phase delay in HIV+ participants [β (95% CI) = 1.21 (1.68); *p* = .006] with no adjusted effect of age (*p* = .379) or sex (*p* = .070).

**Figure 3:**
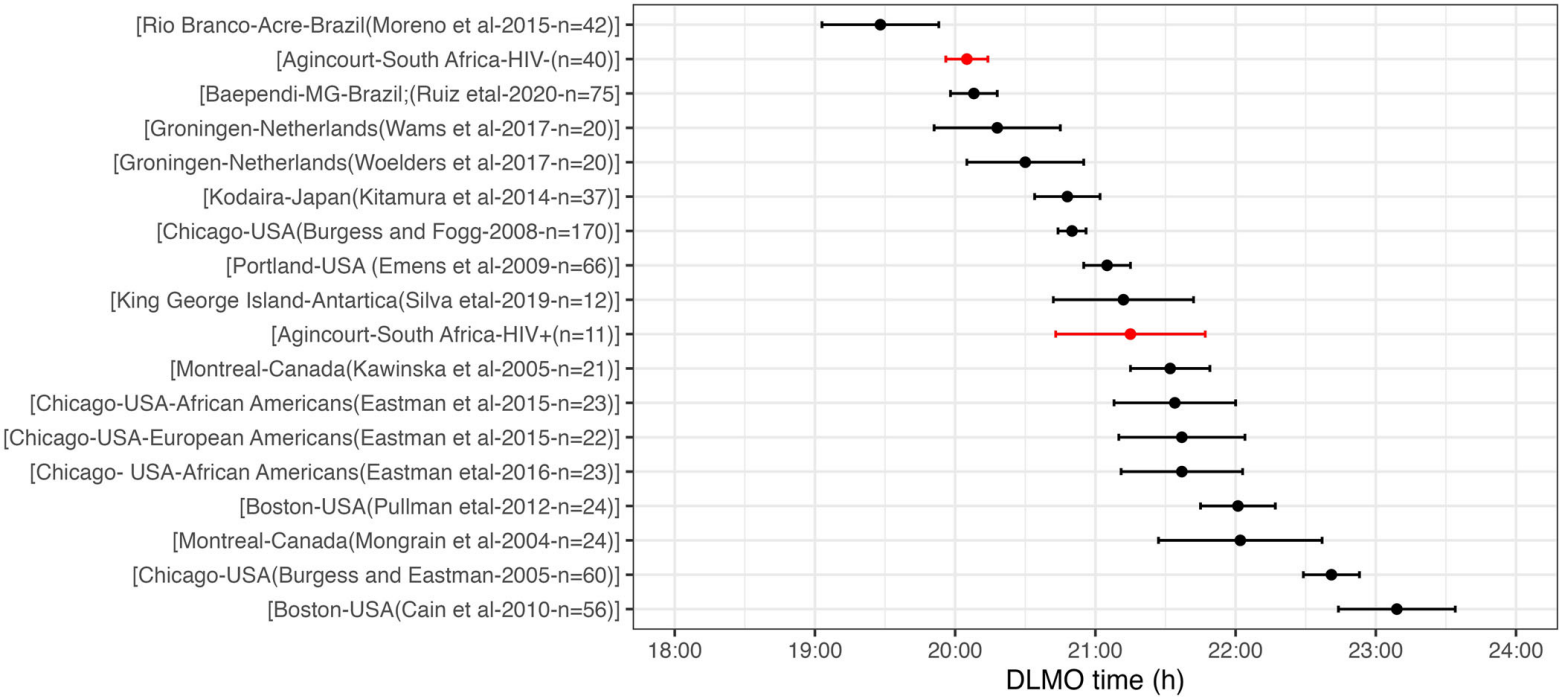
DLMO times for the HIV+ and HIV− participants from the current study (shown in red) compared to published DLMO data from other adult populations. Figure modified from Ruiz et al.^18^ where references are listed. Data are presented as mean ± SEM. DLMO, dim light melatonin onset; HIV, human immunodeficiency virus.

In Figure 3, we extend a previously published figure^18^ displaying DLMO times from 16 other studies to contextualise the timing of DLMO observed in the HIV+ and HIV− participants in the present study in relation to other cohorts.

### 3.3 Phase angle of entrainment

**Figure 4:**
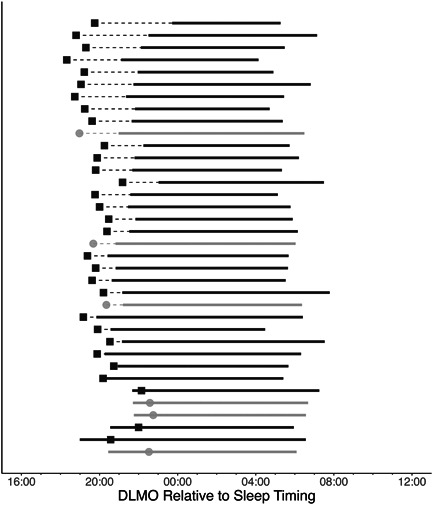
Relationship between DLMO time and habitual actigraphy‐derived sleep timing (*n* = 36). DLMO time is represented by black squares for HIV− participants (*n* = 30) and grey circles for HIV+ participants (*n* = 6). Average actigraphy‐derived sleep onset and offset across the actigraphy collection period were plotted for each participant, represented by the horizontal bars. DLMO, dim light melatonin onset; HIV, human immunodeficiency virus.

**Figure 5:**
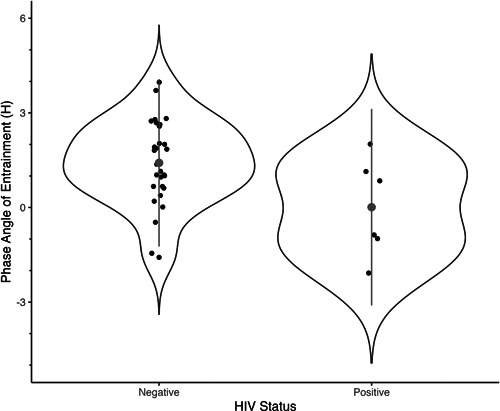
Violin plot comparing the phase angle of entrainment between the HIV− (*n* = 30) and HIV+ (*n* = 6) participants. Individual data points are plotted in addition to the median represented by the larger grey dot, with the dispersion displayed as a violin plot. HIV, human immunodeficiency virus.

Finally, we explored the relationship between DLMO time and habitual actigraphy‐derived sleep onset in our participants as a measure of the phase angle of entrainment between the sleep–wake cycle and endogenous circadian rhythms. Given the relatively small sample for whom we have both valid actigraphy and DLMO data (*n* = 36, HIV+: *n* = 6, 16%), we present these data descriptively in Figure 4, which shows each participant's DLMO time relative to their habitual sleep period and Figure 5, which shows the phase angle of entrainment between the HIV− and HIV+ groups. The observed data suggest that HIV+ participants had a smaller phase angle between DLMO time and habitual sleep onset, with half of the HIV+ group having an earlier habitual sleep onset than DLMO time, suggesting a negative phase angle of entrainment. DLMO time was associated with sleep offset time (Spearman's correlation: *n* = 36, *ρ* = 0.47, *p* = .005) but not with sleep onset time (*n* = 36, *ρ* = −0.086, *p* = .623). When doing partial correlations adjusting for HIV status, we observed the same results.

## 4 DISCUSSION

In this randomly selected and representative sample from an ageing rural South African population, PLWH had significantly later DLMO timing (by more than an hour) compared to those without HIV infection. To contextualise this, the early DLMO timing in the HIV− participants in this study was very close to that observed in a Brazilian Baependi cohort, another rural community in the global south (Figure 3),^18^ while that of the HIV+ group was very much in line with urban cohorts in the United States. In spite of their later circadian phase, however, the timing of sleep onset of PLWH was only slightly delayed (10 min in adjusted analyses) and not correlated with DLMO timing. This would suggest that PLWH are sleeping earlier in their circadian phase compared to HIV− participants, resulting in a shorter phase angle of entrainment between the circadian and the sleep−wake cycle. The possible consequences of a smaller phase angle of entrainment include difficulty in initiating and maintaining sleep, similar to what is observed in younger adults living with insomnia and in healthy older adults whose sleep is more disrupted than that of younger adults.^24^, ^25^ This later timing of DLMO with a shorter phase angle of entrainment may to some extent explain our observation of the slightly shorter total sleep time (12 min in adjusted analyses) derived from actigraphy in PLWH, although we have no way of distinguishing to what extent this shorter total sleep time may also result from inflammatory processes, the infection itself, or comorbid conditions such as pain or depression.^8^, ^26^ Nonetheless, the shorter, potentially mistimed sleep relative to the endogenous circadian cycle observed in this study provides objectively measured evidence supporting the abundant previous subjective reports of poor sleep quality and insomnia in PLWH.^8^, ^26^, ^27^

Sleep efficiency, a marker of sleep quality, was generally low in this population, regardless of HIV status, and was poorer than would generally have been expected in this age group.^28^ This suggests that the sleep environment, in general, was not optimal (a well‐documented feature of sleep in deprived areas of the global south, associated with factors such as sleeping quarters, temperature, noise and concerns about personal safety^19^, ^29^), with similar amounts of time spent awake during the night regardless of HIV status. The fact that PLWH had a significantly shorter sleep time, even though their sleep efficiency was not significantly different, may be partly explained by the slight delay in sleep onset and slight advance in sleep offset observed in the actigraphy data of PLWH. We hypothesise that the advanced age of our sample may make it difficult to see true differences in sleep efficiency between the two groups, which both had age‐related reduced efficiency, and therefore efficiency may not be reduced further by HIV infection. Other limitations include that the DLMO and actigraphy data were based on different (but partially overlapping) subsets of participants as well as the absence of data on napping, light exposure, sleep–wake schedules, sleep and mood disorders, all of which may influence sleep parameters and circadian phase.

Although the timing of seroconversion is not known, most of the ageing PLWH in this study will have lived with the infection for a number of years; for this reason, we cannot entirely rule out the theoretical possibility that our observation reflects some kind of survivor effect driven by delayed circadian phase. The observation does suggest multiple novel lines of research. A key element will be to confirm whether the delayed circadian phase is also present in PLWH of different ages and living in other settings. This is important to understand the biological reason for and downstream effects of this circadian phase delay and its potential roots in abnormal circadian entrainment. A biological cause for an HIV‐related phase delay in circadian rhythms is supported by a mechanistic animal study, which found phase delays of the rest‐activity cycle of mice associated with SCN injection of the HIV protein Tat. In this study, further experiments showed that Tat injection impeded the downstream process of light entrainment in the SCN rhythms.^13^ In a human study, HIV+ participants had higher morning melatonin levels at 09:00, a finding compatible with a delay in circadian rhythms in PLWH.^14^ In that study, the higher morning plasma melatonin levels were associated with higher HIV protein Tat plasma levels, suggesting a direct biological effect of Tat on circadian rhythms in PLWH.

Globally, the relative risk of HIV seroconversion is more than 20 times higher in certain groups (gay men and other men who have sex with men, people who inject drugs, and sex workers) compared to adults aged 15–49 years in the general population.^16^ Behavioural factors associated with belonging to one or more of these groups would be strong potential confounders for studies of sleep and circadian phase. By contrast, in rural Southern Africa, the epidemic has been less demographically discriminating, as is evident from its much higher general population prevalence. This strengthens the biological validity of the delayed circadian phase in PLWH observed in this study. There are no notable differences in lifestyle between the HIV− and HIV+ individuals in this study. The members of this ageing population are mostly beyond retirement age, living quiet, rural lives supported by government remittances and subsistence farming. Potential lifestyle differences would have been much harder to control for if we had studied a younger population or indeed one living on another continent. If the lifestyle‐independent circadian misalignment observed in the current study is confirmed to be a constant feature of chronic HIV infection, then it may be a mediator both of poorer sleep health and of poorer physical health in PLWH, which could potentially be alleviated through light therapy or chronobiotic medication or supplements.

## AUTHOR CONTRIBUTIONS

D. E. Rae, F. X. Gómez‐Olivé, M. von Schantz and K. Scheuermaier designed the study. K. N. Redman, K. E. O'Brien, F. S. Ruiz and K. Scheuermaier developed methods and collected data. K. N. Redman, K. E. O'Brien, F. S. Ruiz, D. E. Rae, F. X. Gómez‐Olivé, M. von Schantz and K. Scheuermaier analysed and interpreted the data. K. N. Redman, K. E. O'Brien, F. S. Ruiz, D. E. Rae, F. X. Gómez‐Olivé, M. von Schantz and K. Scheuermaier prepared the manuscript. All authors approved the final version of the manuscript.

## Data Availability

The data that support the findings of this study are available on request from the corresponding author. The data are not publicly available due to privacy or ethical restrictions.
